# CRISPR/Cas9 genome editing system confirms centriolin’s role in cytokinesis

**DOI:** 10.1186/s13104-021-05898-w

**Published:** 2022-01-10

**Authors:** Eric Seronick, Jae Son, Cameron Michael, Hannah Fogg, Zeynep Gromley, Adam Gromley

**Affiliations:** DeBusk College of Osteopathic Medicine, Lincoln Memorial University, Harrogate, TN 37752 USA

**Keywords:** Centriolin, Centrosome, CRISPR, Cytokinesis

## Abstract

**Objective:**

In addition to its function as the microtubule organizing center of the cell, the centrosome has functions in many other cellular processes including primary cilia formation, DNA damage checkpoints, and cell cycle progression. But the role of individual components of the centrosome in these processes remains unclear. Previous studies used siRNA (small interfering RNA) to “knock down” protein levels of the centrosome component centriolin, resulting in failed cytokinesis. Since this approach was transient, only targeting centriolin at the mRNA level, we sought to confirm these findings by permanently disrupting the gene encoding centriolin using the CRISPR/Cas9 system of genome editing.

**Results:**

This study provides evidence that the CRISPR/Cas9 system is capable of effectively reducing centriolin protein levels in the cell. Furthermore, this disruption leads to a failure of cytokinesis that is reminiscent of the phenotype previously reported for the siRNA-mediated disruption of centriolin. Furthermore, no additional defects in cell division were observed, consistent with results seen with previous siRNA studies. We conclude that the CRISPR/Cas9 system is an effective means of permanently removing the cellular pools of centriolin and that the disruption of centriolin at both the mRNA level and genomic level lead to similar cell division defects.

**Supplementary Information:**

The online version contains supplementary material available at 10.1186/s13104-021-05898-w.

## Introduction

The centrosome is the primary microtubule-organizing center in animal cells, located adjacent to the nucleus during interphase. In mitosis, centrosomes form the poles of the mitotic spindle, which is necessary for proper segregation of duplicated centrosomes and the subsequent formation of two normal, diploid daughter cells. Although the centrosome’s role in the organization of the microtubule network inside the cell has been well established [1], there is significant evidence that components of this organelle may have important functions in many other cellular processes including primary cilia formation [2], DNA damage checkpoints [1], cell cycle progression [3–5], mitotic exit [1, 3, 6], and cytokinesis [6, 7].

Previously it was shown that one of the components of the centrosome, centriolin, has multiple roles in cell division. It was first observed that this protein is necessary for mitotic exit, as disruption results in aberrant cell division and arrest of the cell in the subsequent G1 phase [3]. Later studies determined that centriolin plays a key role in the final abscission event at the end of cytokinesis, acting as a scaffold at the midbody for the recruitment of complexes that control the membrane fusion events necessary for physical separation of the daughter cells [7].

These previous studies utilized siRNA targeting the centriolin mRNA in order to eliminate the protein, but effects of disrupting the gene itself were not investigated. In order to determine if the disruption of the centriolin gene would lead to additional cell division defects, we elected to use the genome editing technology CRISPR/Cas9 to permanently eliminate centriolin and then to assay for any abnormal cell cycle phenotypes that result.

Clustered Regularly Interspaced Short Palindromic Repeats (CRISPR)/Cas9 is a RNA-mediated adaptive immune system identified in both bacteria and archaea that has been modified to allow for specific genome editing in eukaryotes [8]. The CRISPR component of this system includes a guide RNA (gRNA), and the Cas9 is a non-specific CRISPR-associated endonuclease. The gRNA is a short RNA molecule whose sequence is complimentary to a specific target DNA sequence in the genome. In the cell, this gRNA is responsible for bringing the Cas9 endonuclease to a specific genomic region based on its sequence, resulting in a double stranded cut in the DNA and effectively disrupting the expression of the targeted gene. To date, this technology has been used to manipulate the genomes of a wide variety of organisms [9].

In this paper, we describe the abrogation of centriolin function using CRISPR/Cas9. We show that CRISPR/Cas9 can effectively disrupt the production of centriolin protein in Hela cells, and we use immunofluorescence microscopy to show that the centrosomes in these cells are devoid of centriolin. Furthermore, we present evidence that the CRISPR/Cas9-mediated elimination of centriolin causes a cytokinesis defect that is identical to the cytokinesis defect that has been reported previously using siRNA [3]. We conclude that the CRISPR/Cas9 system is an effective means of permanently removing the cellular pools of centriolin and that the disruption of centriolin at both the mRNA level and genomic level lead to similar cell division defects.

## Main text

### Materials and methods

#### Genomic sequence analysis, guide RNA design, and plasmids

Genomic sequence analysis of the *CNTRL* locus was obtained from the UCSC Genome Browser (https://genome.ucsc.edu/cgi-bin/hgGateway). The sequence for the centriolin guide RNA (gRNA) was determined using the MIT CRISPR Design tool (http://crispr.mit.edu/). In order to express the gRNA in the cell, the following single stranded DNA oligos were annealed and cloned into the CRISPR/Cas9 expression vector using Gibson Assembly (New England Biolabs, cat# E2611):

5′-TTTCTTGGCTTTATATATCTTGTGGAAAGGACGAAACACCGCCAATATGAGATCT AGGTCACTT -3′ and

5′-GACTAGCCTTATTTTAACTTGCTATTTCTAGCTCTAAAACAAGTGACCTAGATCTC ATATTGG C

The CRISPR/Cas9 dual-expression vector pSpCas9(BB)-2A-Puro (PX459) was a gift from Feng Zhang (Addgene plasmid # 48139) [10].

#### Cell culture and transfections

Hela cells (ATCC-CCL2) were obtained from ATCC and maintained in Eagle’s Minimum Essential Medium (Catalog No. 30-2003) supplemented with 10% fetal bovine serum following the recommended protocol.

Plasmids were transfected into Hela cells using Turbofect reagent (Thermo Scientific) following the manufacturer’s protocol. Following transfection, the cells were selected for puromycin resistance using 0.5 µg/ml puromycin (Fisher BioReagents).

#### Antibodies, immunofluorescence, and western blotting

Antibodies to the following proteins were used: centriolin (Santa Cruz Biotechnology, cat. # sc-135020), g-tubulin (Invitrogen, cat # MA531482), and α-tubulin (Invitrogen, cat. # 62204). TUNEL assay (Promega, cat # G7360) was performed according to manufacturer’s protocol. For immunofluorescence, cells were fixed in methanol and processed for immunofluorescence as described [11]. All immunofluorescence images were captured on a Nikon Eclipse 50i immunofluorescence microscope equipped with a Nikon DXM1200C digital camera and a 40 × Plan Fluor objective with a 0.75 numerical aperture. Western blotting was performed using standard procedures.

### Results

#### CRISPR/Cas9 targeting of the centriolin gene

**Fig. 1:**
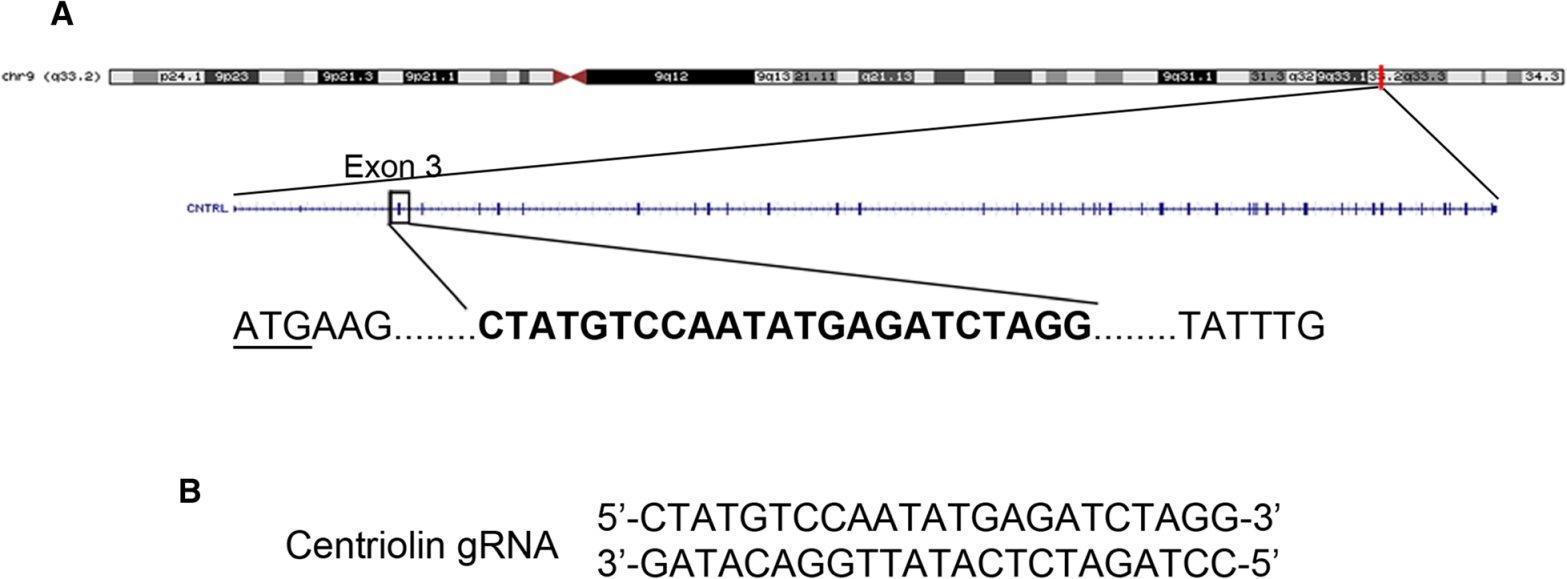
Targeting of the *CNTRL* gene with CRISPR guide RNA. **A** Schematic showing the location of the *CNTRL* gene, represented as a red line, on the long arm of chromosome 9 (9q33.2). The 43 exons comprising the centriolin locus are shown below, along with the specific sequence within exon 3 that was targeted by the guide RNA in bold. The translational start codon is underlined. Adapted from the UCSC Genome Browser graphic. **B** The double-stranded DNA sequence that was cloned into the CRISPR vector is shown

In humans, the gene for centriolin (*CNTRL*) is found on the long arm of chromosome 9 at position 9q33.2 (Fig. 1A). The gene consists of 43 introns and 44 exons, spanning approximately 100 kb. Within exon 3 lies the translational start site for this gene.

In order to disrupt the *CNTRL* gene and prevent the synthesis of functional centriolin within the cell, a guide RNA (gRNA) was designed that targets the third exon (Fig. 1B). The gRNA binds specifically to a sequence that lies 83 nucleotides downstream of centriolin’s translational start site and is immediately upstream from the obligatory Protospacer Adjacent Motif (PAM) sequence [8]. This sequence is highly conserved among vertebrates, being present in nearly all mammalian species for which genomic data is available (data not shown).

The centriolin gRNA sequence was cloned into the dual-expression vector pSpCas9(BB)-2A-Puro, a plasmid containing both a cloning site for the guide RNA as well as an expression cassette in which the *S. pyogenes* Cas9 gene is under control of a CBh (CBA (Chicken Beta-Actin) hybrid) promoter. The CBh promoter is an altered version of the CAG (Promoter consisting of cytomegalovirus (CMV) early enhancer element, the promoter region, the first exon, and the first intron of chicken beta-Actin gene, and the splice acceptor of the rabbit beta-Globin gene) promoter [12] that has been shown to provide robust expression in Hela cells. When expressed in the cell, the *S. pyogenes* Cas9 creates a double strand break at a specific site within the genome determined by the gRNA, resulting in disruption of the targeted gene. This plasmid also contains a puromycin resistance gene, which was subsequently used to select for transfected cells in the presence of puromycin.

#### CRISPR/Cas9-mediated reduction in centriolin protein levels

**Fig. 2:**
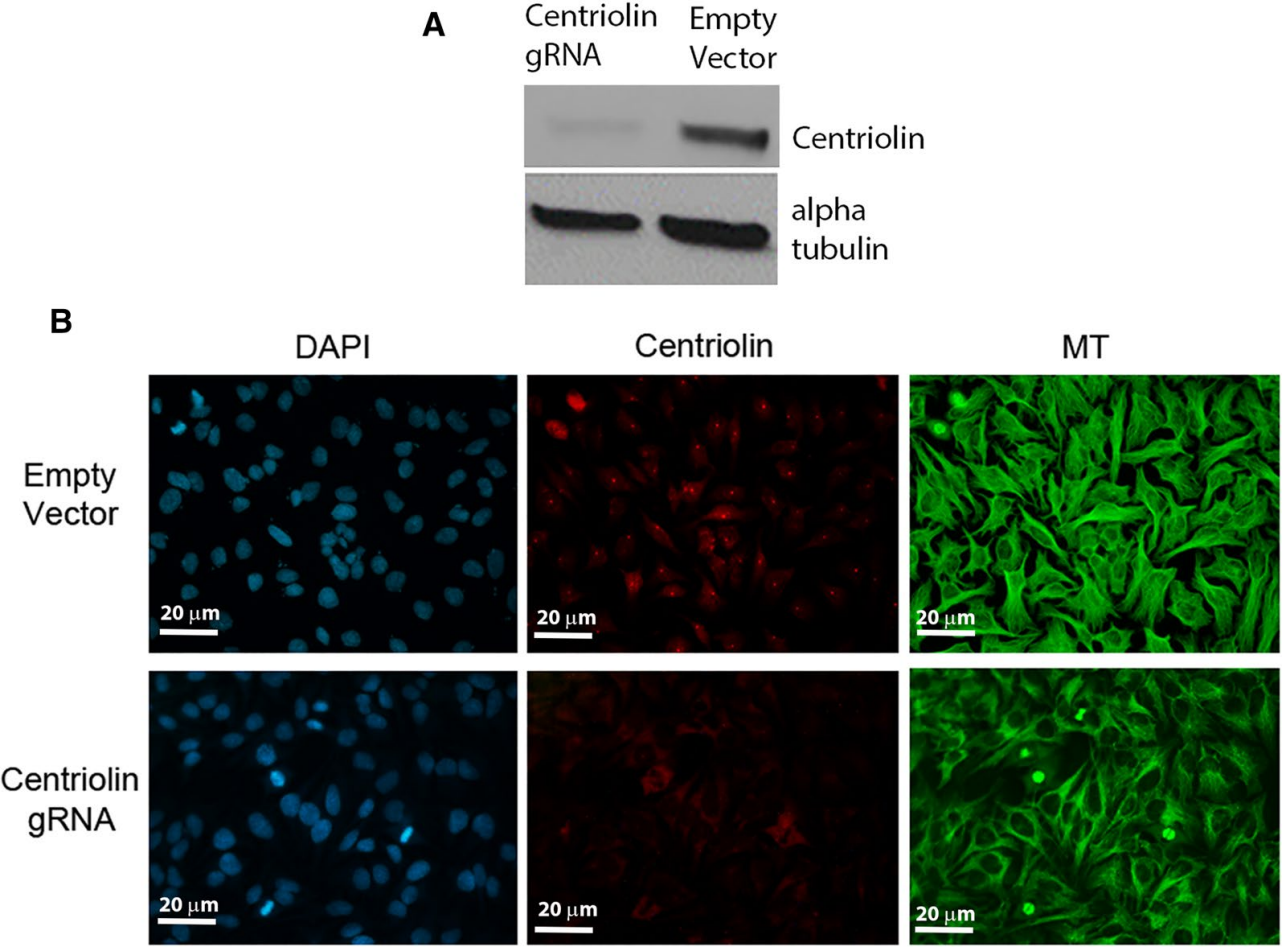
Centriolin is effectively targeted with the centriolin guide RNA and Cas9 in Hela cells. **A** Western blot of whole cell lysates from Hela cells transfected with either the centriolin guide RNA (gRNA)-Cas9 plasmid or empty Cas9 vector and probed with centriolin antibody. Alpha tubulin was used as a loading control. **B** Immunofluorescence staining of centriolin (red) and microtubules (MT, green), and DAPI staining of DNA in centriolin gRNA-treated Hela cells and empty vector controls. Note the brightly stained red dots of centriolin representing the centrosome in the empty vector panel, which are absent in the centriolin gRNA sample. Images were taken at 10× magnification

The pSpCas9(BB)-2A-Puro plasmid containing the centriolin gRNA was transfected into exponentially growing Hela cells. After 48 h, the cells were harvested, and cell lysates were generated. A western blot was performed with the cell lysates using antibodies against centriolin or alpha tubulin (loading control). It was found that in the presence of the centriolin gRNA there was a nearly complete elimination of centriolin protein (Fig. 2A, Additional file 3: Fig. S3). The residual centriolin seen is likely due to the fact that the vector that was used was later found to have a mutation in the puromycin resistance gene (https://www.addgene.org/48139/ see “Depositor Comments” section), reducing the selection efficiency.

The transfected cells were also processed for immunofluorescence with antibodies against centriolin and microtubules. As shown in Fig. 2B, treatment with the centriolin gRNA showed no apparent effect on the microtubule array in the cells. This was true of both interphase and mitotic cells. However, there was a near complete absence of centriolin signal at the centrosomes, proving that the gene disruption achieved was sufficient to eliminate the centrosome-associated pool of centriolin.

#### Loss of centriolin leads to defects in cytokinesis

**Fig. 3:**
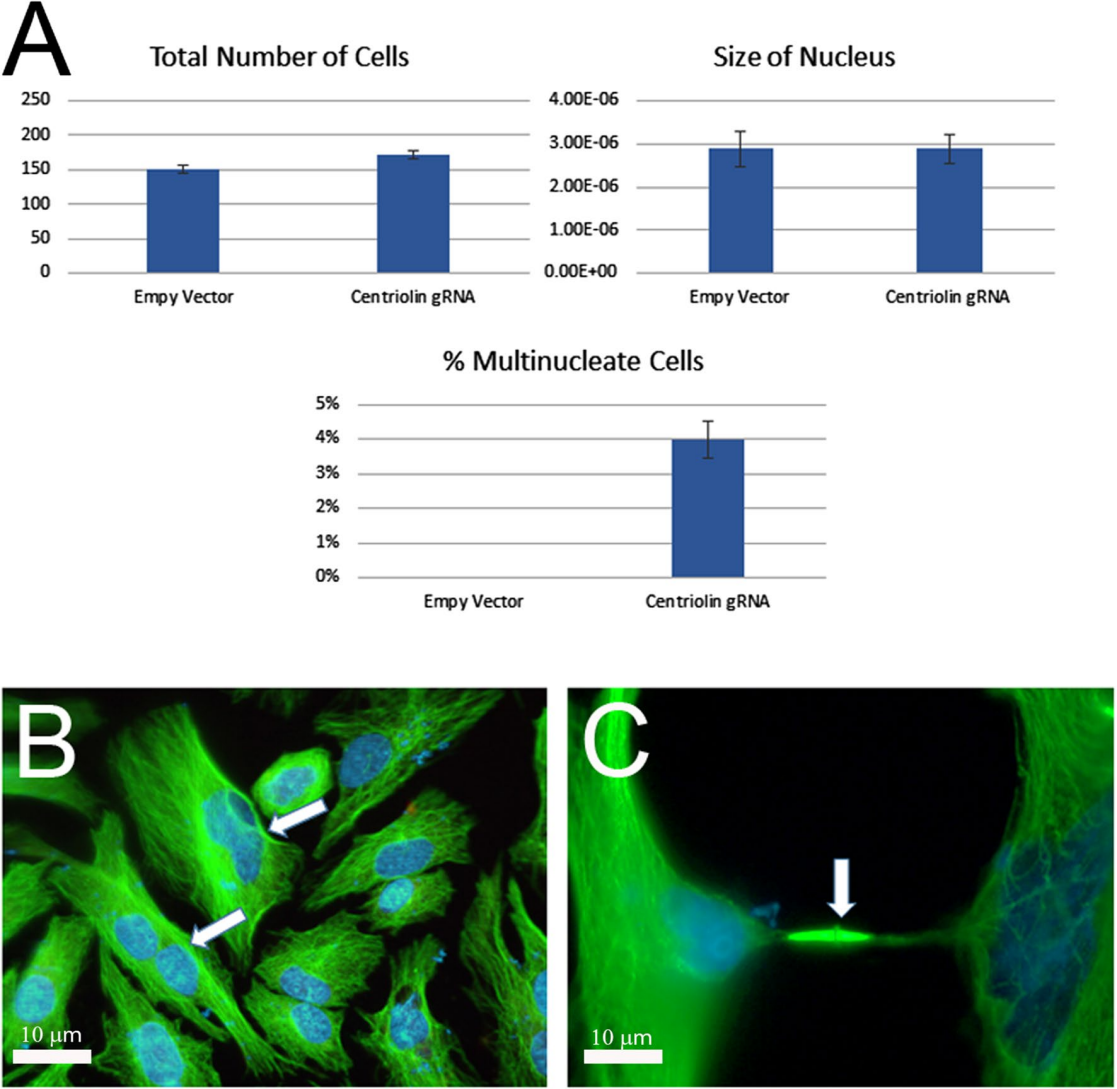
Disruption of the centriolin gene with CRISPR/Cas9 results in the accumulation of multinucleated cells. **A** The number of cells, size of nuclei, and percentage of multinucleated cells was recorded. The data represents three independent experiments. **B** and **C** Immunofluorescence image showing examples of multinucleated cells (**B**, arrows) and daughter cells with a persistent intercellular midbody bridge (**C**, arrow). Blue is DAPI stain and green is microtubules. Images were taken at 40× magnification

The treated Hela cells were analyzed for the presence of any morphological changes. Despite the lack of any disruption in the microtubule organization within the cells, it was noted that there was a marked increase in the percentage of multinucleate cells with the centriolin gRNA treatment, which was not observed in the empty vector controls (Fig. 3A and B). The level of multi-nucleation in these cells is consistent with previous studies that used siRNA to disrupt centriolin [3]. This increase in multinucleated cells did not appear to be due to a difference in the rate of cell death as both the empty vector and the centriolin gRNA populations had similar numbers of total cells (Fig. 3A). In addition, the individual nuclei of the cells in both conditions were of similar size (Fig. 3A), suggesting that the effect on the cells is solely a consequence of a failed late stage of division, rather than a defect in DNA replication or in an earlier mitotic stage. Similar results were seen at 96 h, with a slight increase in the number of multinucleated cells (data not shown). Lack of a dramatic increase at this timepoint is likely due to the inhibition of cell cycle progression upon centriolin disruption that was reported previously [3]. Indeed, cell cycle status indicated by centrosome number revealed a robust arrest in G1 for these cells (Additional file 1: Fig. S1). In addition to the multinucleated cells, a perceptible number of cell pairs connected by persistent intercellular bridges was also seen in the centriolin gRNA samples (Fig. 3C), indicating that the cells were unable to complete the final abscission event in cytokinesis. These results did not appear to be due to any effects associated with DNA damage, as our treated cells were negative for TUNEL staining (Additional file 2: Fig. S2). Notably, our data are consistent with the previously reported phenotypes observed when centriolin protein levels were reduced using siRNA [3].

### Discussion

In this paper, we show that the CRISPR/Cas9 is effective at disrupting the centriolin gene, *CNTRL*. Treatment of cells with Cas9 along with a guide RNA targeting the third exon of *CNTRL* results in a significant reduction in centriolin protein. This loss of centriolin leads to a cytokinesis defect reminiscent of the phenotype previously reported when centriolin levels were reduced with siRNA.

The fact that we did not see a complete loss of centriolin and, hence, a greater penetrance of the cytokinesis defect phenotype, is likely due to an inefficient selection of transfected cells in our experiments. A mutation in the puromycin resistance gene in the pSpCas9(BB)-2A-Puro (PX459) has been reported that reduces the effectiveness of the puromycin resistance gene. This required us to use puromycin during our selections at a much lower concentration than is typically used for Hela cells [13–15]. Therefore, the inefficient elimination of non-transfected cells brought about by these low concentrations of puromycin is most likely responsible for the residual centriolin detected in our western blots. Nevertheless, the phenotype we report here is consistent with previously published studies in which centriolin protein levels were reduced using siRNA. This confirms that the disruption of the *CNTRL* gene by CRISPR/Cas9 affects the cell in the same way as the reduction of centriolin protein by siRNA and indicates that the phenotype we have observed is not due to an off-target effect of the CRISPR/Cas9.

A surprising finding that emerged from previous studies was that centriolin did not have any apparent contribution to the centrosome’s function as a microtubule organizing center [3]. When cells were depleted of centriolin with siRNA, the cells retained an intact microtubule network, which appeared to function normally in both interphase and mitosis. Since centriolin localizes to structures inside the centrosome that are known to be anchoring sites for microtubule minus ends [3], it was expected that elimination of centriolin would adversely affect the cell’s microtubule network. Indeed, we also did not observe any defects in the microtubule network of the cells in our studies. In both our studies as well as those previously published, a complete elimination of the centriolin protein was not achieved. Perhaps a small, residual amount of centriolin is sufficient for its function in organizing microtubules. Future studies are needed to address this possibility.

We have shown that the CRISPR/Cas9 system can be an effective tool for eliminating centriolin, allowing for a more detailed interrogation of its function within the cell. It is worth noting that the sequence that is targeted by our centriolin guide RNA (gRNA) is evolutionarily conserved, being absolutely identical to centriolin homologues in nearly all mammalian species whose genomes have been sequenced to date. Therefore, this gRNA may prove useful in the future to identify centriolin’s cellular function in a multitude of diverse species.

## Limitations

A limitation of our studies is that they were conducted only in Hela cell cultures. It would be important to repeat these experiments in other transformed cell lines, as well as primary cell cultures, to confirm the effects are consistent in other cell types.

## Supplementary Information

**Additional file 1: Fig S1.** Centriolin CRISPR/Cas9 targeted cells arrest in G1. The number of centrosomes in each cell was determined using g-tubulin stain. One centrosome indicates cells in G1, whereas two centrosomes indicate cells in G2. DAPI staining and morphology were used to separate out the mitotic cells within the G2/M group.

**Additional file 2: Fig S2**. Centriolin CRISPR/Cas9 does not induce DNA damage. TUNEL staining was performed on Centriolin CRISPR/Cas9 treated cells and control cells. The percentage of TUNEL positive cells was determined by immunofluorescent microscopy. DNAse treated cells were used as a positive control for the TUNEL assay.

**Additional file 3: Fig S3.** Full Western blot image used to create Fig. 2A. The 48 h post-transfection samples were used to generate the figure.

## Data Availability

The human Centriolin gene (*CNTRL*) sequence used in this study is NCBI GenBank Accession # NM_007018 XM_005251679 XM_352972 https://www.ncbi.nlm.nih.gov/nuccore/NM_007018.6. Analysis of the *CNTRL* locus using the UCSC Genome Browser can be found using the following link https://genome.ucsc.edu/cgi-bin/hgTracks?db=hg38&lastVirtModeType=default&lastVirtModeExtraState=&virtModeType=default&virtMode=0&nonVirtPosition=&position=chr9%3A121074955%2D121177610&hgsid=1237244373_hKLofDAhzAxaDJ6ujBvnGnuADlMg. The CRISPR centriolin guide RNA (gRNA) was determined using the MIT CRISPR Design tool http://crispr.mit.edu/. The CRISPR/Cas9 dual-expression vector pSpCas9(BB)-2A-Puro (PX459) was obtained from Addgene https://www.addgene.org/48139/.

## Abbreviations

gRNA: Guide RNA
CRISPR: Clustered regularly interspaced short palindromic repeats
siRNA: Small interfering RNA
PAM: Protospacer adjacent motif
