# Supplementary figures and images for: CRISPR/Cas9 genome editing system confirms centriolin’s role in cytokinesis
Source: BMC Res Notes. 2022 Jan 10;15:8. doi: 10.1186/s13104-021-05898-w (PMC8751070; doi:10.1186/s13104-021-05898-w)

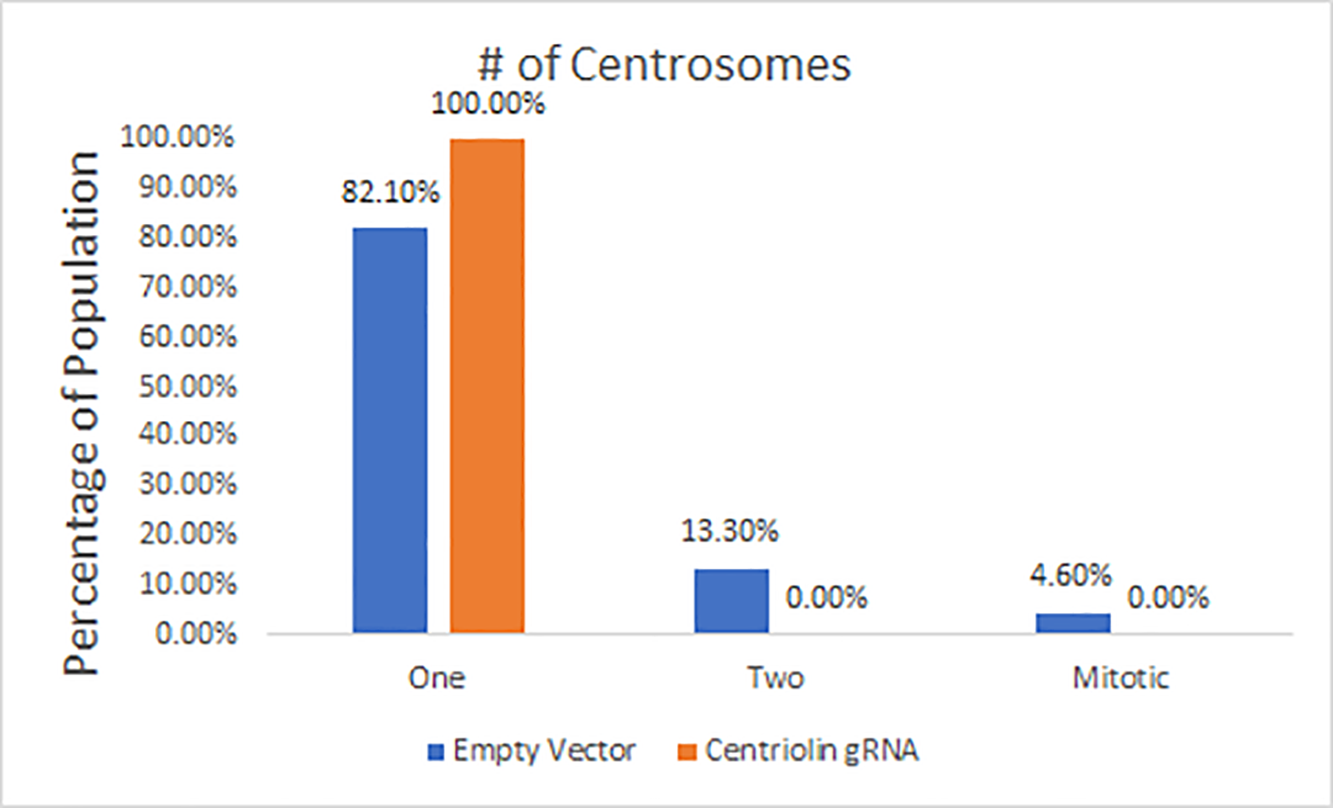

Supplement: Supplementary file 1 — Additional file 1: Fig S1. Centriolin CRISPR/Cas9 targeted cells arrest in G1. The number of centrosomes in each cell was determined using g-tubulin stain. One centrosome indicates cells in G1, whereas two centrosomes indicate cells in G2. DAPI staining and morphology were used to separate out the mitotic cells within the G2/M group. [file 13104_2021_5898_MOESM1_ESM.tif]

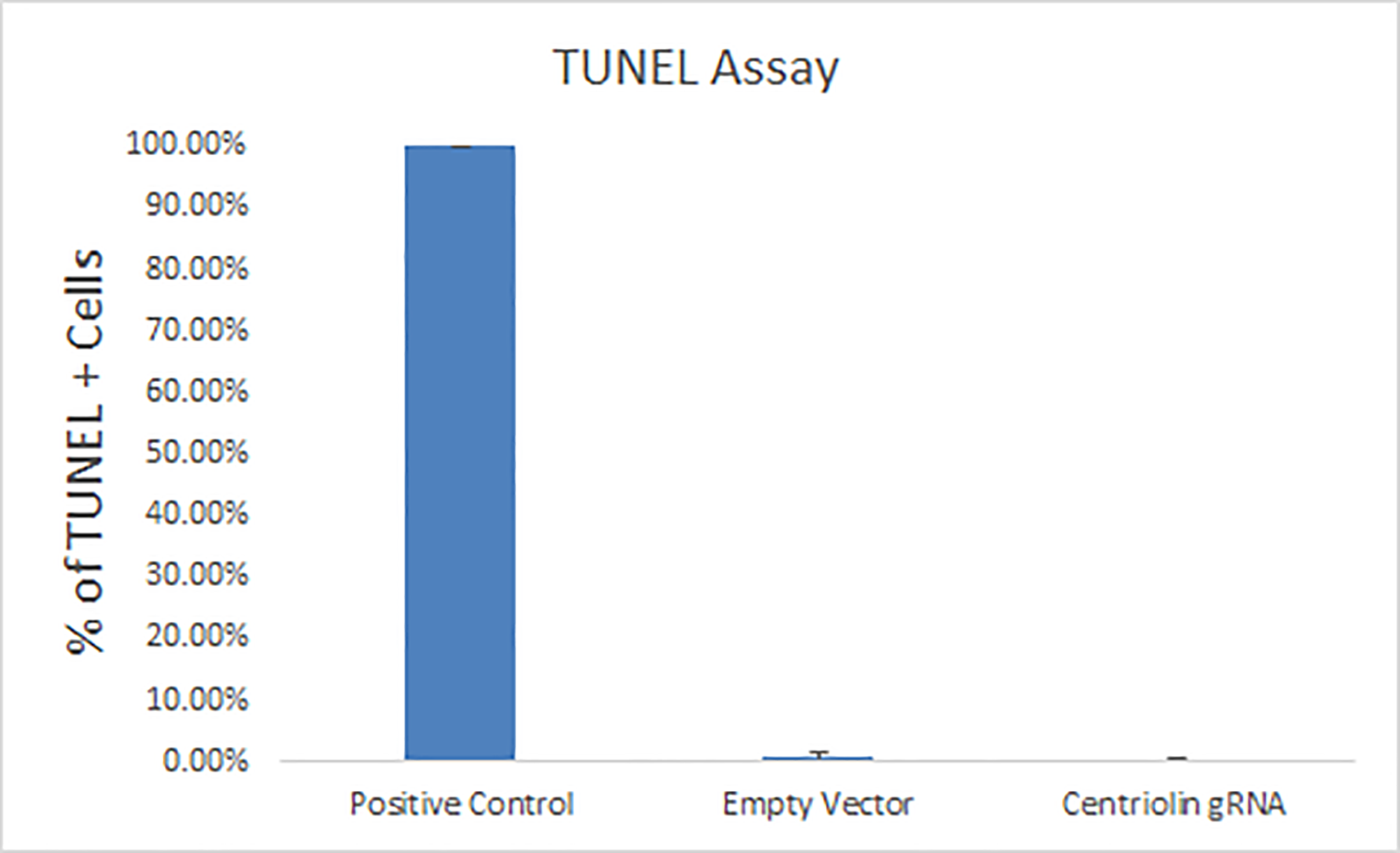

Supplement: Supplementary file 2 — Additional file 2: Fig S2. Centriolin CRISPR/Cas9 does not induce DNA damage. TUNEL staining was performed on Centriolin CRISPR/Cas9 treated cells and control cells. The percentage of TUNEL positive cells was determined by immunofluorescent microscopy. DNAse treated cells were used as a positive control for the TUNEL assay. [file 13104_2021_5898_MOESM2_ESM.tif]

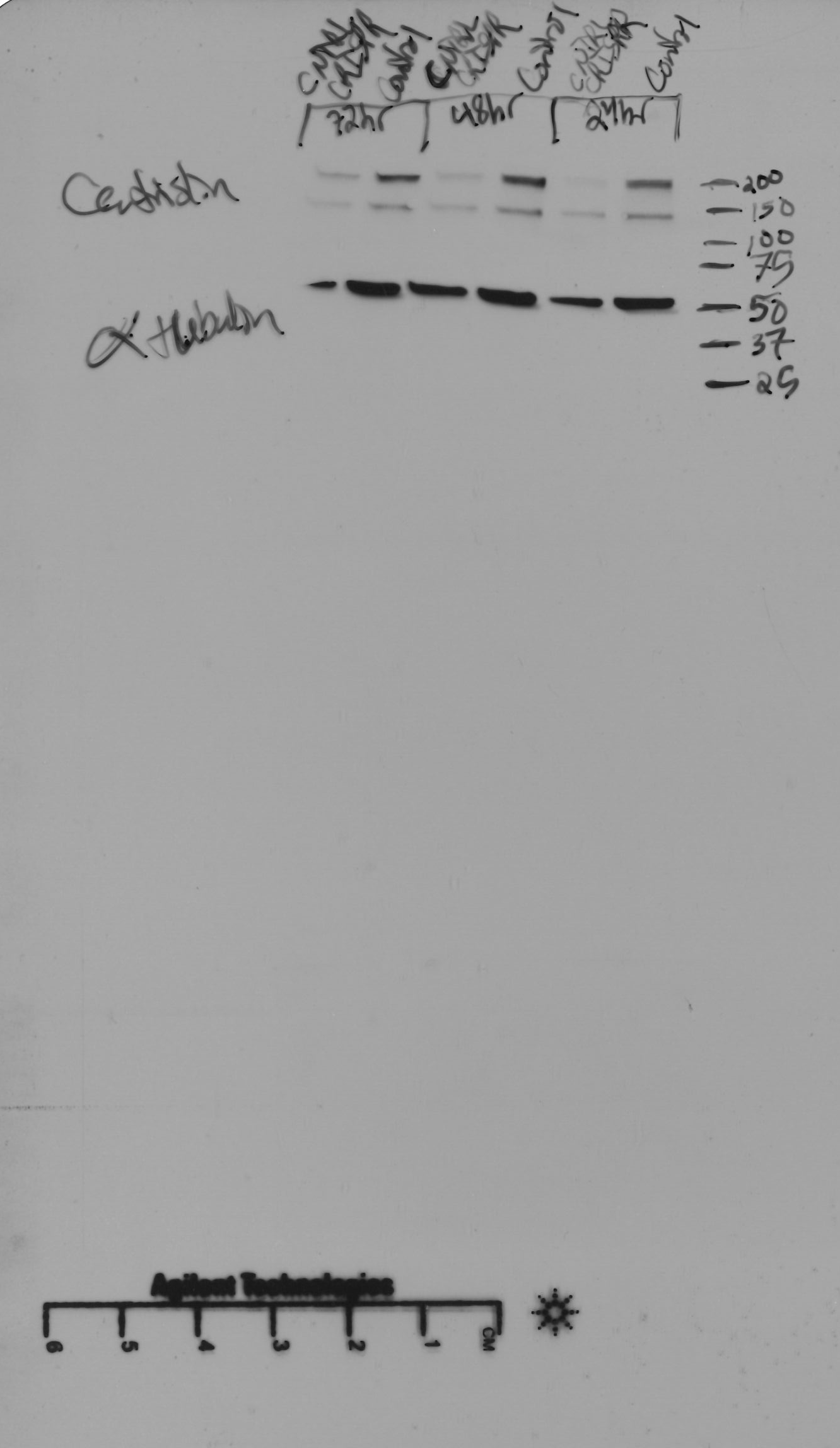

Supplement: Supplementary file 3 — Additional file 3: Fig S3. Full Western blot image used to create Fig. 2A. The 48 h post-transfection samples were used to generate the figure. [file 13104_2021_5898_MOESM3_ESM.tif]
